# The emerging role of fumarate as an oncometabolite

**DOI:** 10.3389/fonc.2012.00085

**Published:** 2012-07-31

**Authors:** Ming Yang, Tomoyoshi Soga, Patrick J. Pollard, Julie Adam

**Affiliations:** ^1^ Henry Wellcome Building for Molecular Physiology, Nuffield Department of Medicine, University of Oxford,Oxford, UK; ^2^ Institute for Advanced Biosciences, Keio University,Tsuruoka, Japan; ^3^ Oxford-Keio Metabolomics Consortium,Oxford, UK; ^4^ Oxford-Keio Metabolomics Consortium,Tsuruoka, Japan

**Keywords:** fumarate, oncometabolite, succination, dysregulated metabolism, mitochondrial dysfunction

## Abstract

The drive to understand how altered cellular metabolism and cancer are linked has caused a paradigm shift in the focus of cancer research. The discovery of a mutated metabolic enzyme, isocitrate dehydrogenase 1, that leads to accumulation of the oncometabolite 2-hydroxyglutarate, provided significant direct evidence that dysfunctional metabolism plays an important role in oncogenesis. Striking parallels exist with the Krebs cycle enzyme fumarate hydratase (*FH*), a tumor suppressor, whose mutation is associated with the development of leiomyomata, renal cysts, and tumors. Loss of FH enzymatic activity results in accumulation of intracellular fumarate which has been proposed to act as a competitive inhibitor of 2-oxoglutarate-dependent oxygenases including the hypoxia-inducible factor (HIF) hydroxylases, thus activating oncogenic HIF pathways. Interestingly, our studies have questioned the role of HIF and have highlighted other candidate mechanisms, in particular the non-enzymatic modification of cysteine residues (succination) that could lead to disruption or loss of protein functions, dysfunctional cell metabolism and cell signaling. Here, we discuss the evidence for proposing fumarate as an onco-metabolite.

## THE LINK BETWEEN DYSREGULATED METABOLISM AND CANCER

Cancer cells exhibit characteristic “hallmarks” of malignancy including increased proliferation, survival, and in particular dysregulated metabolism (Hanahan and Weinberg, 2011). There is abundant evidence showing that cancer cells produce energy through a high rate of glycolysis in the cytoplasm, in marked contrast to the process in most normal cells, which employ a relatively low rate of glycolysis followed by oxidation of pyruvate in the mitochondria (Kim and Dang, 2006). Although Otto Warburg postulated that this switch in cellular metabolism was the fundamental cause of cancer, most cancer research since has focused on mutations in, and roles of, oncogenes and tumor suppressors in the onset and progression of cancers (Warburg et al., 1927; Warburg, 1956; Semenza et al., 2001; Vander Heiden et al., 2009). The development and application of highly sensitive new technologies such as mass spectrometry and nuclear magnetic resonance combined with metabolic labeling and profiling have increased our understanding of the complexities of normal and dysregulated cellular metabolism, particularly when linked with powerful computing programs that allow for the integration and interrogation of data(Tomita and Kami, 2012). Furthermore, cancer associated mutations have been identified in genes of known metabolic function; namely isocitrate dehydrogenase 1 and 2 (IDH1 and 2), succinate dehydrogenase (SDH) and fumarate hydratase (FH; Semenza, 2011). Consequently, there has been renewed interest in Warburg’s hypothesis and the link between dysregulated metabolism and cancer.

## WHAT IS AN ONCOMETABOLITE?

The term oncometabolite has only recently been coined and assigned with confidence to *R*(-)-2-hydroxyglutarate ((*R*)-2HG), the reduced form of 2-oxoglutarate (2OG). (*R*)-2HG is a by-product produced by gain-of-function mutations of IDH1 and IDH2, which normally catalyze the reversible NADP^+^-dependent oxidative-decarboxylation of isocitrate to produce 2OG in the cytoplasm and mitochondria, respectively (Leonardi et al., 2012). IDH mutations have been found in 75% of low grade gliomas and secondary glioblastoma multiforme and approximately 20% of acute myeloid leukemia (Parsons et al., 2008; Mardis et al., 2009; Yan et al., 2009). 2HG acts as a competitive inhibitor to multiple 2OG utilizing 2-oxygenases, including prolyl hydroxylases (PHDs), histone demethylases, and the TET family of 5-methylcytosine (5mC) hydroxylases (Chowdhury et al., 2011; Xu et al., 2011). In gliomas, (*R*)-2HG accumulation caused by oncogenic IDH mutations enhances DNA methylation and epigenetic remodeling, which stalls cell differentiation and thereby primes cells for malignancy (Figueroa et al., 2010; Ward et al., 2010; Lu et al., 2012).

How should we define an oncometabolite? Using (*R*)-2HG as an example, one could propose that an oncometabolite is a small molecule component (or enantiomer) of normal metabolism whose accumulation causes metabolic dysregulation and consequently primes cells allowing future progression to cancer. There are likely to be numerous and complex interacting steps in this process including inhibition, disruption or activation of pathways each of which will require detailed investigation. Nevertheless, the concept of oncometabolites is novel and exciting and offers a real and innovative route into therapies for a variety of cancers. Here we will discuss evidence implicating fumarate as an oncometabolite in FH-deficient cells. Furthermore, we will highlight where these studies have provided useful insights into cell metabolism.

## FUMARATE HYDRATASE

Germline loss-of-function mutations in the Krebs cycle enzyme FH predispose affected individuals to benign cutaneous and uterine leiomyomata, renal cysts and aggressive collecting duct and Type 2 papillary renal tumors in hereditary leiomyomatosis and renal cell cancer (HLRCC; Tomlinson et al., 2002). However, the exact mechanisms leading to FH-associated oncogenesis remain to be elucidated (Frezza et al., 2011a).

**FIGURE 1:**
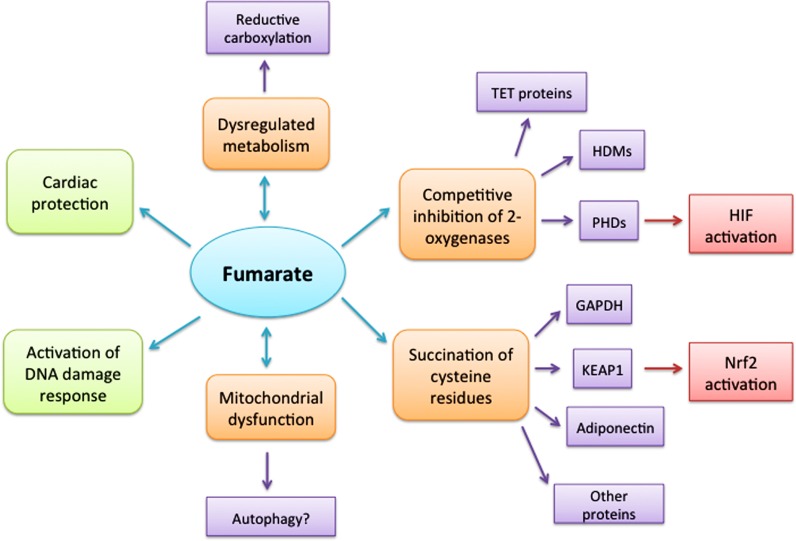
**Consequences of elevated cellular fumarate.** Loss of fumarate hydratase enzyme activity results in intracellular accumulation of fumarate with multiple diverse consequences. However, it remains to be determined whether some, or all of these, or indeed other as yet uncovered pathways, lead directly to oncogenesis. Dysregulated metabolism possibly linked to reductive carboxylation may both result from elevated fumarate and is certainly a cause of the elevated fumarate. Mitochondrial dysfunction is a feature of both altered metabolism and possibly high fumarate levels; but whether it is a contributing factor in oncogenesis needs to be determined and if autophagy leads to increased availability of nutrients for the cell. Fumarate has been shown to act as a competitive inhibitor of members of the 2-oxoglutarate-dependent oxygenase superfamily including the histone demethylase enzymes (HDMs), TET proteins and hypoxia-inducible factor (HIF) hydroxylases, thus activating oncogenic HIF pathways. However, further investigation is required to ascertain whether fumarate initiates oncogenesis *via* all, or any, of these routes. Succination of cysteine residues that could lead to disruption or loss of protein functions, dysfunctional cell metabolism and cell signaling offers a novel and promising route to link fumarate and oncogenesis directly. The benefits of fumarate proposed in activating a DNA damage response need to be addressed further, while the cytoprotective role proposed for fumarate in cardiac cells by diverting amino acids into the Krebs cycle and activating the Nrf2 antioxidant pathway suggests that different cell types may have different response strategies.

Fumarate hydratase is a highly conserved homotetrameric protein located and functioning in both the mitochondria and the cytosol. In the mitochondria, FH catalyses the hydration of fumarate to generate malate as part of the Krebs cycle. This pathway is not only essential for the production of cellular energy, but also forms a central metabolic hub to generate macromolecular precursors. In the cytosol, FH has been proposed to participate in a number of pathways where fumarate can be produced, including the urea cycle and the purine nucleotide cycle (Stepinski et al., 1989; Brosnan and Brosnan, 2004). Both forms of FH are encoded by the same transcript; localization of the protein is effected by cleavage of the resulting propeptide into two smaller peptides, one retaining the N-terminal mitochondrial targeting sequence (MTS) and one that is released into the cytoplasm (Stein et al., 1994; Sass et al., 2001). How FH is localized within the cell and the exact role the enzyme plays in different cellular compartments have not been elucidated fully and this will certainly be a focus for future research. Currently, it is unclear whether the mitochondrial Krebs cycle defect is responsible for oncogenesis, or if other mechanisms contribute, such as fumarate accumulation (**Figure 1**). To address this question we have used a conditional *Fh1* (the ortholog of human FH) knockout mouse model (Pollard et al., 2007) and a panel of four mouse embryonic cell lines (MEFs) derived from this: wild-type MEFs, Fh1-deficient MEFs (Fh1^-^^/^^-^, Fh1KO), and Fh1-deficient MEFs in which there is stable re-expression of either full length, mitochondrial-targeted FH (Fh1^-^^/^^-^ + FH), or cytoplasmic FH (Fh1^-^^/^^-^ + FHΔMTS; O’Flaherty et al., 2010). These have been used to investigate the importance of FH in both the mitochondria and the cytoplasm and to unravel some of the complex consequences of FH loss for cellular, tissue and whole animal physiology with successful extrapolation into FH-deficient human tumors (O’Flaherty et al., 2010; Adam et al., 2011). Immunofluorescence studies with the MEFs described above have demonstrated that Fh1 loss results in a striking change in the morphology of mitochondria, which become much enlarged (O’Flaherty et al., 2010). This phenotype reinforces the observation that mitochondrial dysfunction is associated with FH deficiency; but the precise reasons for this and the consequences for the mitochondria and the cell remain to be determined. It could be postulated that disruption to the Krebs cycle leads to alterations in mitochondrial membrane potential and permeability of the outer membrane and increased autophagy; all aspects of cell biology and physiology that can, and should, be investigated.

## FUMARATE ACCUMULATION – A CONSEQUENCE OF FH INACTIVATION

Fumarate hydratase-deficient cells and tumors have been shown to accumulate fumarate to very high levels with multiple consequences including the activation of oncogenic pathways (Isaacs et al., 2005; Pollard et al., 2005). In Fh1 deficient MEFs the level of fumarate is approximately 8–10 fmol/cell as measured by ^1^H magnetic resonance spectroscopy metabolite analysis and no fumarate can be detected by this technique in either wild-type MEFs or Fh1^-^^/^^-^ + FH MEFs (O’Flaherty et al., 2010). Perhaps surprisingly only very low levels (approximately 1 fmol/cell) can be detected in Fh1-deficient MEFs complemented with extramitochondrial FH (Fh1^-^^/^^-^ + FHΔMTS), although the defect in aerobic metabolism is not corrected (O’Flaherty et al., 2010). Currently, we are undertaking metabolomic analyses to confirm these observations in MEFs by alternative techniques (capillary electrophoresis time-of-flight mass spectrometry; Soga et al., 2003, 2006) and to extend the studies to mouse and human tissues lacking FH. It would be interesting to determine the relative levels of fumarate under a variety of physiological conditions in different cellular compartments; mitochondrial *versus* cytoplasm – especially since cytoplasmic “rescue” effects such a dramatic reduction in the overall cellular fumarate levels (O’Flaherty et al., 2010) and in the nucleus, given the proposed role for FH in the DNA damage response in yeast (Yogev et al., 2010).

## COMPETITIVE INHIBITION OF 2-OXOGLUTARATE-DEPENDENT OXYGENASES

Others had postulated previously that FH-associated tumorigenesis might be driven by the upregulation of a number of oncogenic pathways by hypoxia inducible factor (HIF; Gottlieb and Tomlinson, 2005). Indeed, it has been shown that fumarate competitively inhibits 2OG-dependent oxygenases, particularly the HIF PHDs, thus mimicking hypoxia (pseudohypoxia), stabilizing the HIF complex and potentially activating its oncogenic target genes (Isaacs et al., 2005).

Hypoxia inducible factor is stabilized in human tumors in HLRCC, in Fh1-deficient MEFs and in the hyperplastic renal cysts that develop in mice following targeted inactivation of Fh1. Gene expression analysis in all these tissues revealed strong signatures of HIF activation (Isaacs et al., 2005; Pollard et al., 2005, 2007; Ashrafian et al., 2010). Furthermore, both succinate and fumarate inhibit PHD enzymatic activities *in vitro* and cell-permeable esters of 2OG reactivate the enzymatic activity of the PHDs and alleviate the pseudohypoxia caused by succinate or fumarate accumulation (Hewitson et al., 2007; Mackenzie et al., 2007). However, using a mouse model in which Fh1 inactivation in renal tubular cells was combined with inactivation of Hif-1α, Hif-2α, or both Hif-α isoforms; hyperplastic cyst formation was shown to be Hif independent (and separately Phd independent). Indeed combined inactivation of Fh1 and Hif-1α greatly exacerbated the cystic hyperplasia (Adam et al., 2011). While this suggests that the effect of HIF may be discounted in the early events of fumarate-mediated oncogenesis it neither precludes a role in tumorigenesis for long-term stabilization of HIF nor its consequent activation of multiple oncogenic pathways.

This is by no means an end to the story as recent evidence has shown that fumarate (and succinate) inhibit the activity or function of other members of the 2OG oxygenase superfamily, including histone demethylase enzymes (HDMs) and TET proteins which are critical in epigenetic regulation of gene expression (Xiao et al., 2012). Despite the identification of cancer-associated mutations in both classes of these enzymes, a direct causal role in oncogenesis is yet to be determined (Abdel-Wahab et al., 2009; van Haaften et al., 2009; Dalgliesh et al., 2010; Ko et al., 2010).

## SUCCINATION

In addition to its role as an allosteric regulator of 2OG-dependent oxygenases, fumarate is also an endogenous electrophile and reacts spontaneously with cysteine residues in proteins by a Michael addition reaction to form *S*-(2-succinyl) cysteine (2SC), a process termed succination (Alderson et al., 2006). Accumulation of cellular fumarate has been shown to correlate directly with an increase in succinated proteins. It has been proposed that this results from mitochondrial stress in adipocytes during adipogenesis, when cultured in high glucose medium, in adipose tissue of obese type 2 diabetic mice and in skeletal muscle of streptozotocin-induced type 1 diabetic rats (Frizzell et al., 2011). Mechanistically, it has been proposed that nutrient excess from hyperglycemia results in high a NADH/NAD^+^ ratio, leading to feedback inhibition of oxidative phosphorylation and accumulation of mitochondrial intermediates including fumarate, which in turn causes protein succination (Frizzell et al., 2012). Targets for succination include the glycolytic enzyme glyceraldehyde-3-phosphate dehydrogenase, adiponectin, cytoskeletal proteins, and endoplasmic reticulum chaperone proteins. Furthermore, evidence suggests that succination of these proteins in cells may impair their functions (Blatnik et al., 2008; Frizzell et al., 2009).

Compared to the situation in diabetes, protein succination is predictably more severe in FH-deficiency due to the significantly higher levels of fumarate accumulation. Immunohistochemical analysis of FH-deficient tumors and cysts has shown a striking direct relationship between *FH* inactivation and an increase in 2SC proteins, which is absent in non-HLRCC tumors and normal tissue controls and has provided a potentially robust diagnostic biomarker for FH-deficiency in cells and tissues (Bardella et al., 2011). We hypothesize that succination resulting from FH deficiency targets multiple proteins and may, at least in part, account for the altered metabolism and oncogenic drive observed in HLRCC, as exemplified by the succination of Kelch-like ECH-associated protein 1 (KEAP1). Evidence for KEAP1 succination came from the observation that there is striking upregulation of the nuclear factor (erythroid-derived 2)-like 2 (NRF2)-mediated antioxidant signaling pathway in our murine Fh1 deficient renal cyst model, mouse embryonic fibroblasts as well as human FH-deficient cells and tissues (Adam et al., 2011; Ooi et al., 2011). NRF2 controls the adaptive response of cells to oxidative and electrophilic stress, through the activation of target genes containing antioxidant response elements (AREs) while KEAP1 is the substrate recognition subunit of a Cul3-based E3 ubiquitin ligase complex and a major cellular electrophile sensor (Zhang, 2010). In the absence of electrophiles, the homodimeric KEAP1 interacts with an NRF2 monomer, promoting its ubiquitylation and proteasomal-mediated degradation (McMahon et al., 2010). KEAP1 has been shown to be succinated on two critical cysteine residues (Cys155 and Cys288) in FH-deficient cells, which disrupts its interaction with NRF2, resulting in stabilization and accumulation of nuclear NRF2 (Adam et al., 2011; Ooi et al., 2011). This allows binding to AREs and consequent activation of downstream target genes involved in defense against reactive oxygen species (Zhang, 2010). The activation of the NRF2-mediated antioxidant pathway is a clear point of focus for future work; especially as activating *NRF2* mutations and inactivating *KEAP1* mutations are prevalent in many cancer types (Hayes and McMahon, 2009) and oncogene-induced Nrf2 transcription promotes tumorigenesis in mice (DeNicola et al., 2011). NRF2 may contribute to tumor development by enabling FH-deficient cells to tolerate high levels of exogenous or endogenous oxidants, thus promoting their survival.

Succination may result in the disrupted function of multiple proteins and offers a unique mechanism by which fumarate may lead to dysregulated cellular metabolism and act as an oncometabolite. Clearly screens need to be undertaken to identify other candidate succination targets which have cysteine residues critical for their function and are associated with oncogenic signaling or metabolic pathways.

## DISRUPTION TO METABOLISM

The Krebs cycle dysfunction caused by loss of FH activity poses significant challenges to cells in meeting energy requirements, in the generation of macromolecular precursors and in survival. Studies, in part contradictory, using a number of cellular models, have identified a variety of mechanisms by which FH-deficient cells may deal with these problems. Impaired respiration and upregulation of aerobic glycolysis have been observed in FH-deficient cell lines and tissues, presumably as an adaptation to meet cellular energy requirements by producing ATP independently of the TCA cycle (Sudarshan et al., 2009; O’Flaherty et al., 2010). Elevated glutaminolysis has been observed and stable isotope labeling studies of an Fh1-deficient murine renal cell line have suggested that glutamine is the major carbon source for the Krebs cycle (Frezza et al., 2011b). These authors have also proposed upregulation of the heme biosynthesis pathway as a means of removing excess carbon from the dysregulated Krebs cycle whilst permitting partial mitochondrial NADH generation (Frezza et al., 2011b). Enhanced glycolysis and glutaminolysis are both stereotypical features of transformed cells (DeBerardinis et al., 2007; Vander Heiden et al., 2009) and may prime FH-deficient cells toward malignancy. Separately, partial reversal of the Krebs cycle, so called glutamine-dependent reductive carboxylation, has been observed in human carcinoma lines including UOK262 cells, deficient in FH. By this mechanism 2OG is reductively carboxylated by IDH isoforms to generate isocitrate, followed by its subsequent metabolism to produce citrate, oxaloacetate and acetyl coenzyme A (AcCoA). AcCoA is crucial for fatty acid synthesis and protein acetylation while oxaloacetate is reduced to malate to compensate for decrease in the levels of these metabolites due to Krebs cycle blockage (Metallo et al., 2012; Mullen et al., 2012). Such a mechanism would allow cells with FH deficiency and impaired oxidative phosphorylation to maintain cell growth. There are some anomalies between these various proposed adaptive responses perhaps relating to the cellular model systems employed. More comparable analyses need to be conducted, ideally both *in vitro* and *in vivo* and in combination with metabolite and transcriptome profiling. Clearly, however, the adaptive response to fumarate accumulation of FH-deficient cells through alterations of primary metabolism may contribute to oncogenic transformation. Glycolysis, glutaminolysis, anaplerosis and the urea cycle may all be relevant for FH-deficiency and a greater understanding of these and their inter-relationships in normal and dysregulated cell metabolism are vital.

## CELL-SPECIFIC EFFECTS – A DUAL ROLE FOR FUMARATE

Despite evidence for fumarate as an oncometabolite, in other circumstances this metabolite has been shown to exhibit cytoprotective roles. For example, it has been reported that in yeast cytoplasmic FH translocates to the nucleus following DNA damage. There it activates the damage response to double strand breaks, a process that can be complemented by high concentrations of fumarate in the absence of FH enzymatic activity. Elevated nuclear FH has also been detected in HeLa cells following irradiation damage suggesting that human cytosolic FH may have a similar function (Yogev et al., 2010). Additionally, it has also been shown that elevated fumarate in Fh1 cardiac knockout mice greatly reduced the amount of heart tissue damage following ischemic-reperfusion injury. This is achieved by diverting amino acids into the Krebs cycle, thus maintaining ATP levels, stabilizing Nrf2 and consequently activating the Nrf2 antioxidant pathway (Ashrafian et al., 2012). Perhaps the key to the opposing roles of fumarate as an oncometabolite or in a protective role lies in the exact cellular concentrations of fumarate and its cellular compartmentalization, as this metabolite on its own is essential for normal functioning of the Krebs cycle. Therefore, it would be of immense value to be able to determine the endogenous levels of fumarate in different cells and under different stress conditions. Technical difficulties to undertaking this include heterogeneity in tissue samples and, more significantly, the lack of effective methods to accurately quantify small molecule metabolites such as succinate and fumarate in sub-cellular compartments, e.g., mitochondria and nucleus, where local metabolite levels could be important.

The shift in focus of cancer research to one of trying to understand how altered cellular metabolism and cancer are linked has highlighted how woefully ignorant we are about the complexities and interrelationships of cellular metabolic pathways and how these are altered under conditions of a variety of stress agents. However, studies into rare genetic disorders associated with metabolism are beginning to provide real insights into the adaptive responses of cells and dysregulated metabolism associated with cancer.

## Conflict of Interest Statement

The authors declare that the research was conducted in the absence of any commercial or financial relationships that could be construed as a potential conflict of interest.
